# Integrating HIV services in an era of global change

**DOI:** 10.1002/jia2.70087

**Published:** 2026-03-09

**Authors:** Jirair Ratevosian, Linda‐Gail Bekker, Robyn Eakle, Stefan Baral, Javier Cepeda, Lara Dugas, Mark Dybul, George Alleyne, Serge Paul Eholie, Kene Esom, Anna Grimsrud, Diane Havlir, Adeeba Kamarulzaman, Parastu Kasaie, Michel Kazatchkine, Nduku Kilonzo, Michael Klag, Marina Klein, Sharon Lewin, Chewe Luo, Keletso Makofane, Natasha Martin, Kenneth Mayer, Gregorio Millett, Ntobeko Ntusi, Loyce Pace, Peter Piot, Birgit Poniatowski, Demetre Daskalakis, Anton Pozniak, Thomas Quinn, Carolyn Reynolds, Jürgen Rockstroh, Serra Sippel, Bruno Spire, Ann Starrs, Steffanie Strathdee, Mauro Schechter, Nicholas Thomson, Peter Vickerman, Brian Weir, Chris Beyrer

**Affiliations:** ^1^ Duke Global Health Institute Durham North Carolina USA; ^2^ Desmond Tutu HIV Centre University of Cape Town South Africa; ^3^ Trinity University Washington DC USA; ^4^ Program for Implementation and Equity Research, Department of Epidemiology Johns Hopkins Bloomberg School of Public Health Baltimore MD USA; ^5^ Department of Epidemiology Johns Hopkins Bloomberg School of Public Health Baltimore MD USA; ^6^ Public Health Sciences Loyola University Chicago Maywood Illinois USA; ^7^ Division of Epidemiology and Biostatistics, School of Public Health University of Cape Town Cape Town South Africa; ^8^ Department of Medicine & Center for Global Health Practice and Impact Georgetown University Medical Center Washington DC USA; ^9^ Pan American Health Organization University of the West Indies Bridgetown, Barbados Washington DC USA; ^10^ Department of Dermatology & Infectious Diseases, UFR des Sciences Médicales Université Félix Houphouët‐Boigny Treichville University Teaching Hospital Abidjan Côte d'Ivoire; ^11^ Centre for Global Health Law University of Warwick UK; ^12^ International AIDS Society (IAS) Geneva Switzerland; ^13^ Division of HIV, Infectious Diseases & Global Medicine, Zuckerberg San Francisco General Hospital, Department of Medicine University of California San Francisco San Francisco California USA; ^14^ Monash University Malaysia Subang Jaya Malaysia; ^15^ Global Health Center the Geneva Graduate Institute Geneva Switzerland; ^16^ National AIDS Control Council (NACC) Nairobi Kenya; ^17^ Division of Infectious Diseases & Chronic Viral Illness Service, McGill University Health Centre, Faculty of Medicine McGill University Montreal Ontario Canada; ^18^ Doherty Institute for Infection & Immunity University of Melbourne Melbourne Victoria Australia; ^19^ Africa CDC Addis Ababa Ethiopia; ^20^ Department of Epidemiology Harvard T.H. Chan School of Public Health New York NY USA; ^21^ Division of Infectious Diseases & Global Public Health University of California San Diego San Diego California USA; ^22^ Fenway Institute Harvard Medical School Boston Massachusetts USA; ^23^ amfAR – The Foundation for AIDS Research Washington DC USA; ^24^ Department of Medicine University of Cape Town & Groote Schuur Hospital Cape Town South Africa; ^25^ Meharry Medical College Nashville Tennessee USA; ^26^ London School of Hygiene & Tropical Medicine London UK; ^27^ KU Leuven Leuven Belgium; ^28^ International AIDS Society (IAS) Geneva Switzerland; ^29^ Callen‐Lorde Community Health Center New York New York USA; ^30^ Department of HIV Medicine Chelsea & Westminster Hospital LSHTM London UK; ^31^ Division of Infectious Diseases and Center for Global Health Johns Hopkins University Baltimore MD USA; ^32^ Center for Strategic and International Studies (CSIS) Washington DC USA; ^33^ Department of Medicine University of Bonn Bonn Germany; ^34^ Executive Director The Brigid Alliance New York NY USA; ^35^ Aix Marseille Univ, Inserm, IRD, SESSTIM Sciences Economiques & Sociales de la Santé & Traitement de l'Information Médicale ISSPAM Marseille France; ^36^ Kinaura Partners San Francisco California USA; ^37^ University of California San Diego San Diego California USA; ^38^ Departamento de Doenças Infecciosas e Parasitárias Universidade Federal do Rio de Janeiro Brazil; ^39^ Crawford School of Public Policy Australian National University Australia; ^40^ Population Health Sciences University of Bristol Bristol UK; ^41^ Department of Health, Behavior & Society Johns Hopkins Bloomberg School of Public Health Baltimore MD USA; ^42^ Duke Global Health Institute Durham North Carolina USA

**Keywords:** integration, sustainability, human rights, implementation science, equity, health systems

## 1 INTRODUCTION

In 2018, the International AIDS Society (IAS) Lancet Commission examined the integration of HIV services into broader health systems as a pathway to achieving sustainable health outcomes and advancing the Sustainable Development Goals [1]. Since then, a series of shocks—including post‐COVID‐19 fiscal contractions, geopolitical instability, major U.S. government aid retrenchment, and rapid biomedical and digital innovations—have made integration less a long‐term aspiration and more an operational necessity.

The 2018 Commission also highlighted the vulnerability of vertical HIV programming and funding, maintaining the importance of disease‐specific expertise while simultaneously leveraging HIV investments to strengthen broader health systems [2]. Yet, as a result of many variables—including the COVID‐19 pandemic, new innovations, and shifting global priorities and declining resources for vertical programmes—the move towards more integrated, sustainable health services has significantly progressed.

In the most recent decades of the HIV response, the pandemic has shifted to a chronic condition, and many countries have adopted models that integrate HIV care with broader chronic disease management programmes. People living with HIV face a heightened risk of non‐communicable diseases (NCDs) due to factors like chronic infection, inflammation and metabolic dysfunction associated with older antiretroviral therapy (ART) regimens and ageing. South Africa has leveraged HIV programming to integrate NCD care into other services, including the nationwide Central Chronic Medicines Dispensing and Distribution programme, and medical adherence clubs in Cape Town [3, 4, 5].

The future of an integrated global HIV response is being shaped by converging epidemiological, funding, geopolitical and population‐level shifts that demand urgent adaptation to sustain progress and maintain political support. In early 2025, overnight cuts made by the U.S. government to foreign aid, such as President's Emergency Plan for AIDS Relief (PEPFAR), combined with dismantling of the U.S. Agency for International Development (USAID), reductions by other donors, as well as rising political instability, have caused catastrophic disruptions to funding and HIV services [1, 6, 7, 8].

This sudden upheaval has weakened health systems and curtailed the health workforce, increasing the global risk of being unable to respond effectively to HIV and future pandemics. At the same time, scientific and technological breakthroughs—from long‐acting HIV prevention and treatment to digital and artificial intelligence (AI)‐enabled service delivery—create new opportunities to redesign care in ways that are more efficient, resilient and people‐centred. Against this backdrop, this paper examines four major, inter‐related forces shaping the next phase of HIV integration and assesses what they mean for the future of the global HIV response, universal health coverage schemes, and sustained political and financial commitment to sustain impact and drive progress.

## 2 DISCUSSION

### 2.1 Political polarization

National and international political dynamics are increasingly shaped by conservative and nationalistic trends, presenting significant challenges to the sustainability of global health governance and funding. In donor countries central to the international HIV response—including the United States, France, the United Kingdom, Germany and the Netherlands—shifts towards more isolationist policies have jeopardized commitments to global health initiatives, which have also been affected by the war in Ukraine and financial requirements of growing defence budgets. Since 2015, overall resources for global health have been in decline, and this trend is likely to be exacerbated by these political shifts [9].

In 2025, the U.S. government's initial freeze and limited waiver on PEPFAR funding disrupted services for millions and underscored the fragility of political support for global health initiatives [7]. The lack of reauthorization for PEPFAR, coupled with increased restrictions on LGBTQ+ rights in parts of Eastern Europe and Africa, further threaten the inclusivity of health systems and risk marginalizing key populations. At the same time, the America First Global Health Strategy, released in September 2025, reframes HIV integration by prioritizing bilateral agreements, domestic co‐financing and private sector partnerships over traditional multilateral mechanisms, reshaping accountability and equity in the global HIV response [10].

The U.S. withdrawal from the WHO, and diminished support for other United Nations (UN) agencies like Joint United Nations Programme on HIV/AIDS (UNAIDS) and United Nations Population Fund (UNFPA), have severely undermined global health coordination between countries and other funding mechanisms [11]. This withdrawal will likely disrupt ongoing HIV initiatives and fragment health governance. It may also embolden other nations to deprioritize multilateral engagement on health, undermining global health security, impeding progress towards public health goals and increasing inefficiencies rather than generating cost savings [7, 11]. The global goal of eliminating HIV as a public health threat by 2030 was already slipping out of reach before 2025, and the current shocks now push the response into a period of profound uncertainty where even the status of progress may become increasingly difficult to track [12].

### 2.2 Geographic shifts in HIV acquisitions

Since the beginning of the HIV pandemic, the world has made great strides in scaling up treatment and reducing new acquisitions [13]. According to UNAIDS, there were an estimated 40.8 million people living with HIV in 2024 and a 40% decline in new acquisitions from 2010 to 2023 [13]. However, while some countries have achieved the UNAIDS 95‐95‐95 testing and treatment targets, many more are struggling to reach those goals and still others are seeing their epidemics go in the other direction [14]. The majority of people living with HIV globally reside in eastern and southern Africa, where decades of community leadership, innovation and investment have driven substantial progress [14]. The region has seen dramatic reductions in vertical HIV transmission and expanded ART access, yet the epidemic remains deeply entrenched, particularly among adolescent girls and young women who account for a disproportionate share of new acquisitions [15]. High HIV prevalence among key populations, coupled with structural inequalities, stigma and under‐resourced health systems, continue to fuel the epidemic [16].

Rising acquisitions are particularly evident in eastern Europe, central Asia, Latin America, the Middle East and North Africa, and countries like the Philippines [14]. These regions face a combination of structural barriers, including inadequate national or donor funding for key population programming, challenging legal environments, fractured health systems and entrenched stigma; underscoring the urgent need for context‐driven interventions in these regions [17].

For progress in the HIV response to advance, localized strategies tailored to the social, legal and economic contexts of the regions are imperative. In contexts where same‐sex relationships, drug use or sex work are criminalized, forcing HIV services into mainstream health systems can expose people to stigma, surveillance or arrest. In such settings, stand‐alone, community‐led programmes may remain the most effective and ethical approach, yet their historic reliance on external donors leaves the most marginalized populations at acute risk as funding recedes. This moment necessitates the development of new delivery mechanisms that bundle services and the cultivation of new partners and donors to ensure that services for criminalized and stigmatized populations not only survive but evolve sustainably to meet changing realities. Increased funding, policy reform, discreet digital technologies to reach and link people to services, and robust international collaboration will be key to reversing these alarming trends and ensuring that no population is left behind [14].

### 2.3 Changing paradigms for health governance

The COVID‐19 pandemic profoundly disrupted health systems, with HIV programmes experiencing significant setbacks as resources were diverted to pandemic response efforts [18, 19]. The pandemic also deepened disparities between high‐income countries and low‐ and middle‐income countries (LMICs), exposing profound inequalities in access to vaccines, medical commodities and financial resources [18]. The reallocation of resources threatened to undermine progress in HIV prevention and treatment, but also presented a significant silver lining in terms of opportunities for adaptation. The African Union and African Centres for Disease Control (Africa CDC) took a larger role in solutions for the continent, as opposed to waiting on partnerships with international donors to dictate steps [20]. The constraints on the supply chains for health on importations also accelerated discussions on scaling up regional manufacturing [20].

The creation of the Pandemic Fund and the WHO Pandemic Hub, amendments to the International Health Regulations, and the conclusion of the international negotiations on a WHO Pandemic Agreement and the African Union's granting of autonomous public health agency status to the Africa CDC were also positive steps forward to bolster global health security [21, 22, 23, 24, 25]. Africa, though particularly disadvantaged during the COVID‐19 vaccine rollout, demonstrated remarkable resilience, making progress in establishing new regional health security mechanisms through the Africa CDC, creating an mRNA hub and helping to shape the emerging global health architecture in meaningful ways [20].

Alongside these changes, African leaders have for several years pursued self‐reliance of its HIV response [26, 27]. Rather than relying on donors, African leaders and institutions are promoting health sovereignty through concrete measures: expanding regional manufacturing capacity, strengthening African medicines security, increasing domestic financing and embedding community accountability in governance. Building on the African Union's Agenda 2063, this approach reframes HIV not as a donor‐driven programme, but as part of a broader strategy for resilient health systems [26]. In 2025, “The Accra Reset” reinforced these commitments, setting out pathways for pooled procurement, integrated HIV and health financing, and inclusive decision‐making rooted in African priorities [28]. These directions echo recent recommendations from African policymakers and experts, who stress the urgency of integrating HIV services into national systems, diversifying financing and protecting community‐led models to sustain progress amid donor withdrawal [29].

### 2.4 Technological and service delivery innovations

Since the 2018 IAS Lancet Commission, major scientific advancements have transformed HIV prevention and treatment, including long‐acting medicines. Innovations such as injectable treatments, vaginal rings, long‐acting pre‐exposure prophylaxis (PrEP) and ART, and multipurpose prevention technologies offer new options for those struggling with daily regimens [30, 31]. Additionally, AI‐powered digital health tools provide personalized risk assessments and virtual support, which may furth enhance access and use [32, 33]. These breakthroughs represent a significant step forward in global HIV care and prevention.

Digital health encompasses electronic medical records, m‐health (i.e. use of mobile personal devices for HIV prevention and care), telehealth and machine learning with an emphasis on AI [33]. The ubiquity of mobile devices, particularly mobile phones, has resulted in a multiplicity of technological solutions such as virtual HIV‐specific interventions using chatbots for PrEP and ART counselling; automated text messaging or reminder calls to support ART; and social media platforms to estimate population sizes of key populations in various countries [34, 35, 36]. In addition to telehealth, which flourished during COVID‐19 lockdowns [37, 38], much of the recent growth in the health technology space is in machine learning and AI. Both machine learning and AI have found a foothold in HIV prevention and care [39]. Availability of each of these and future HIV prevention and care innovations will be influenced by political will, policy adaptation, funding, licensing agreements and other factors, none of which are insurmountable.

In addition to technological advances, there are also service delivery innovations that can help diversify and extend person‐centred care. Pharmacy‐based delivery of HIV services has been expanding in recent years, and offers existing infrastructure already trusted across populations for HIV and other primary care, leaving more complex care for traditional clinics [40]. Looking ahead, AI‐powered tools could reimagine HIV prevention by discreetly identifying individuals at elevated risk and linking them to services in confidential, non‐discriminatory ways that complement existing delivery models [41].

## 3 CONCLUSIONS

The HIV response is racing against the clock—and against global headwinds that are increasingly hostile to equity, multilateral cooperation and foreign aid. The future of the HIV response, and global health more broadly, will depend on the ability of countries to rapidly adapt to the major trends driving the global challenges and to seize this moment of disruption as an opportunity for bold, necessary reforms outlined here (see Table 1). HIV service integration will be an important aspect of this adaptation, and integration solutions should take into account the global trends highlighted in this paper. The sudden halt of PEPFAR funding in 2025 and broader donor retrenchment have further amplified these risks, forcing countries to make difficult trade‐offs that could erode prevention and weaken the very programmes needed to sustain epidemic control [42]. As HIV services are increasingly integrated into broader health systems, there is an urgent need for standardized metrics to measure the impact of integration on prevention, treatment outcomes, equity, and system resilience across diverse epidemiologic and financing contexts.

**Table 1:** Key recommendations for the future of the HIV response

Strategic priority	Recommendation
Strengthen HIV service integration	Accelerate the embedding and integration of HIV services into national health systems as a core strategy for sustainability and resilience. Integration must protect and build upon the unique strengths of the HIV response—such as community leadership, rights‐based approaches and stigma reduction—while enhancing broader health system capacity.
Reform governance	Shift to locally driven models that centre country leadership, reduce donor dependency and improve coordination among global health institutions through consolidation to streamline overlapping mandates, align funding and restore trust in multilateralism.
Accelerate HIV donor transitions for sustainability	Support countries in managing accelerated donor transitions by strengthening domestic financing, fostering public‐private partnerships and embedding HIV services into national health systems to ensure long‐term programme viability.
Prioritize equity and community leadership	Embed community‐led monitoring and services in national systems, protect key population programmes, and address legal and social barriers to care.
Measure and recalibrate HIV integration efforts	Systematically evaluate the impact of integrated HIV service delivery on health outcomes, equity and sustainability, and use these findings to guide adaptive targets and policy decisions amid changing epidemiology, financing and global health threats.
Build out additional financing mechanisms	While continued development assistance remains indispensable, it is critical to diversify multilateral funding by leveraging development banks, design innovative mechanisms to introduce and scale up next‐generation prevention tools, and expand pooled procurement.
Invest in R&D and implementation science	Advance innovation in prevention and treatment (e.g. long‐acting options, vaccines, cures and AI tools), and evaluate cost‐effective, integrated delivery models.

Previously, we emphasized the need for reforms that empower LMICs to lead their health responses [2]. This includes shifting from donor‐driven models to more localized decision‐making, strengthening public sector capacity and embedding community engagement in health systems [1]. Additionally, we called for rethinking global health governance. As multiple agencies like the Global Fund, Gavi, UNAIDS and other UN agencies operate with overlapping mandates and fragmented financing, serious questions are emerging about the efficiency of maintaining multiple separate institutions in an increasingly resource‐constrained environment. The Lusaka Agenda underscores this urgency—calling for a more coherent and country‐led global health architecture, built on trust, alignment and shared accountability [43]. Without these reforms, global health risks further fragmentation, undermining equity in treatment and prevention.

A newly formed long‐term view for the future of the HIV pandemic is needed, given that current goals are unlikely to be achieved. This view would take into account the trends outlined here and set new, aspirational but achievable goals. Considerations for HIV service integration within the context of other future pandemics, the need for further localization amidst accelerated donor withdrawal, creative funding mechanisms and leveraging innovation should factor into new goals. As international health priorities expand to address climate change, antimicrobial resistance and NCDs, HIV's position in global funding debates will continue to face challenges unless globally coordinated solutions are in place. A recent *Lancet* Viewpoint cautions that integration under fiscal duress risks eroding quality unless guided by deliberate policy and leadership, highlighting both the fragility of global HIV financing and the opportunity to embed HIV within resilient, person‐centred health systems [44].

In conjunction with global governance is the need for more sustainable, resilient and creative funding mechanisms. PEPFAR and the Global Fund have been successful platforms that have advanced HIV and broader global health programming, adapted to support pandemic responses, and can play a broader role in health security. Leveraging multilateral development banks, development finance institutions and security assistance could offer new avenues for health investments. LMICs could also explore a pooled supply chain regional resourcing to lower costs through bulk procurements. This would help bring down the costs of new interventions, such as long‐acting options for HIV prevention, that require bulk manufacturing and purchasing to reduce pricing.

As highlighted in the Kigali Declaration, adopted at the 13th International AIDS Society Conference on HIV Science in July 2025, continuing support for research and development—grounded in scientific independence—as well as implementation research for evaluating integrated service delivery models, cost‐effectiveness and digital health innovations, is essential to further optimize services and reduce costs in the longer term [45]. There is a pipeline of HIV prevention and treatment drugs, as well as research into vaccines and even cures, that still hold immense promise and should not be abandoned [46, 47]. Finally, as HIV services are increasingly integrated into broader health systems, it is critical to preserve the core principles that have made the HIV response successful: rights‐based approaches, community leadership, transparency and a focus on the most marginalized populations.

## COMPETING INTERESTS

No conflicts of interest to declare.

## AUTHORS’ CONTRIBUTIONS

L‐GB, CB and JR conceptualized and drafted the manuscript. GM, RE, AS, CR, PP, MK, LD and SB contributed substantial sections and provided critical revisions. All authors reviewed, edited and approved the final version of the manuscript.

## FUNDING

No grant funding was utilized for this paper.

## Data Availability

Data sharing is not applicable to this article as no datasets were generated or analysed during the current study.
